# Clique-Based Clustering of Correlated SNPs in a Gene Can Improve Performance of Gene-Based Multi-Bin Linear Combination Test

**DOI:** 10.1155/2015/852341

**Published:** 2015-08-04

**Authors:** Yun Joo Yoo, Sun Ah Kim, Shelley B. Bull

**Affiliations:** ^1^Department of Mathematics Education, Seoul National University, Seoul 151-742, Republic of Korea; ^2^Interdisciplinary Program in Bioinformatics, Seoul National University, Seoul 151-742, Republic of Korea; ^3^Prosserman Centre for Health Research, The Lunenfeld-Tanenbaum Research Institute of Mount Sinai Hospital, Toronto, ON, Canada M5T 3L9; ^4^Division of Biostatistics, Dalla Lana School of Public Health, University of Toronto, Toronto, ON, Canada M5T 3M7

## Abstract

Gene-based analysis of multiple single nucleotide polymorphisms (SNPs) in a gene region is an alternative to single SNP analysis. The multi-bin linear combination test (MLC) proposed in previous studies utilizes the correlation among SNPs within a gene to construct a gene-based global test. SNPs are partitioned into clusters of highly correlated SNPs, and the MLC test statistic quadratically combines linear combination statistics constructed for each cluster. The test has degrees of freedom equal to the number of clusters and can be more powerful than a fully quadratic or fully linear test statistic. In this study, we develop a new SNP clustering algorithm designed to find cliques, which are complete subnetworks of SNPs with all pairwise correlations above a threshold. We evaluate the performance of the MLC test using the clique-based CLQ algorithm versus using the tag-SNP-based LDSelect algorithm. In our numerical power calculations we observed that the two clustering algorithms produce identical clusters about 40~60% of the time, yielding similar power on average. However, because the CLQ algorithm tends to produce smaller clusters with stronger positive correlation, the MLC test is less likely to be affected by the occurrence of opposing signs in the individual SNP effect coefficients.

## 1. Introduction

Current genetic association studies aim to identify genetic variants responsible for a disease by investigating associations between single nucleotide polymorphisms (SNPs) and a trait of interest. In a single-SNP approach, each SNP is analyzed individually for the marginal association with the trait. In a multi-SNP approach, a group of SNPs is analyzed together for polygenic model analysis or gene-based analysis to obtain a global statistic for the combined effect of a set of SNPs [1–8]. When the gene is the unit of interest in the association analysis, gene-based analyses can be performed with multimarker methods using multi-SNP genotypes or haplotypes [6, 8–10]. These methods have the potential benefits of reducing genome-wide type I error burden and boosting the power of the study [9].

Most popular multimarker methods have been developed for the analysis of genotypes. In some methods, the marginal effects of individual SNPs are combined to form a global statistic [5, 11]. In others, SNP genotypes are analyzed in a multiple regression model and global statistics are constructed to represent the joint effects of multiple SNPs in a gene [3, 8, 12, 13]. Some multimarker tests such as C-alpha [14], SKAT [11], and CMC tests [15] specifically target rare variants with minor allele frequency (MAF) less than 1%. On the other hand, multimarker tests such as SKAT-C [16] and the test by Curtis [10] can be applied to a combined set of rare (MAF < 1%), low frequency (1% ≤ MAF < 5%), and common (MAF ≥ 5%) variants.

Multimarker methods can be roughly divided into two types: linear and quadratic tests [17]. Linear tests are constructed by combining the individual SNP effects in a linear combination with certain weights [2, 4, 18]. Linear tests can be powerful if the individual SNP effects have the same direction but can lose substantial power if this condition is not met [2, 5, 8, 17]. Since the direction of an effect can be reversed by recoding the genotype, some researchers developed methods to recode the risk and base alleles considering magnitude and direction of pairwise correlations between SNPs [2, 5]. Quadratic tests are constructed as a quadratic form of an effect vector with corresponding weight matrix [5, 11, 16]. Quadratic tests are more robust to the occurrence of effects in opposing directions, but the degrees of freedom can be high if many neutral SNPs are included in the analysis [13].

Yoo et al. [8, 13] proposed the multi-bin linear combination test (MLC), which is a hybrid of linear and quadratic tests, and evaluated its performance for common SNPs [13] and for combinations of common and low frequency SNPs [8]. For the MLC test, SNPs are partitioned into bins or clusters of highly correlated SNPs according to the pairwise linkage disequilibrium (LD) measure *r*. Then percluster linear combinations of individual SNP effects are combined in a quadratic form [8, 13]. Because of the quadratic component, the MLC test is more robust than linear tests and can have better power than a quadratic test such as the generalized Wald test under realistic causal model scenarios [8, 13].

For SNP clustering, Yoo et al. [8, 13] previously applied an algorithm incorporated in the LDSelect program [19]. LDSelect is designed to select tag SNPs and the cluster partitioning of a gene proceeds by identifying SNPs that capture the effects of other SNPs through LD. Because its greedy algorithm begins with a SNP in LD with the largest number of other SNPs, it tends to first construct one large cluster. However, for the MLC test, clusters with fewer SNPs are less likely to include causal effects in opposing directions and may therefore be more robust. Yoo et al. also showed that the power of the MLC test is better when correlations between SNPs within a cluster are large and positive [13].

In this study, we develop a new clustering algorithm called CLQ that identifies cliques in the network of SNPs. By definition, all pairwise correlations between SNPs in a clique are above a prespecified threshold value. We also incorporate the coding correction algorithm of Wang and Elston [2, 5] into CLQ so that, after recoding, the cliques consist only of positively correlated SNPs. We compare the performance of MLC tests using the previous clustering algorithm, LDSelect, with that using the new CLQ algorithm in terms of power and robustness. For power calculations, we use genotype data from the HapMap Asian population to provide 1000 different realistic LD structures. For one causal and two causal SNP scenarios, we consider all possible causal SNP choices within each gene. Through extensive numerical power calculations for the MLC test under various causal-gene SNP-trait model scenarios, we show that the CLQ algorithm is highly suitable for incorporation into the MLC test.

## 2. Methods and Materials

### 2.1. Multi-Bin Linear Combination Test

Suppose *m* SNPs in a gene are jointly analyzed in a multiple regression. We denote the genotypes of *m* SNPs as *X*_1_, *X*_2_,…, *X*_*m*_. The genotypes can be coded differently depending on the genetic model. For the rest of the paper, we assume an additive genetic model such that *X*_*i*_ is the count of minor alleles for *i*th SNP; that is, *X*_*i*_ = 0, 1, or 2. We set up the regression model as

(1) $$\begin{array}{c} g^{-1}\left\{E\left(Y\right)\right\}=\beta_{0}+\beta_{1}X_{1}+\beta_{2}X_{2}+\cdots +\beta_{m}X_{m}, \end{array}$$

where *g* ^−1^(·) is the link function. For the global hypothesis of gene-based association, we construct a test using the estimated beta coefficients, $\hat{\beta }=({\hat{\beta }}_{1},{\hat{\beta }}_{2},\ldots ,{\hat{\beta }}_{m})$, and the covariance matrix of $\hat{\beta }$, Σ.

Suppose *m* SNPs are partitioned into several bins or clusters based on the pairwise linkage disequilibrium measure *r* defined as

(2) $$\begin{array}{c} r_{ij}=\frac{P_{ij}-P_{i}P_{j}}{\sqrt{P_{i}P_{j}\left(1-P_{i}\right)\left(1-P_{j}\right)}}, \end{array}$$

where *P*_*i*_ and *P*_*j*_ are the MAF values of the *i*th and *j*th SNP and *P*_*ij*_ is the frequency of the haplotype consisting of the two minor alleles. If phase information of genotypes to identify haplotypes is not available, *P*_*ij*_ is estimated using maximum likelihood methods, which is the same as computing the Pearson correlation coefficient *r* between additive genotypes *X*_*i*_ and *X*_*j*_. If *m* SNPs are partitioned into *l* clusters, we construct a *m* × *l* matrix *J* to denote SNP assignments such that *J*_*ij*_ = 1 if the *i*th SNP belongs to the *j*th cluster and *J*_*ij*_ = 0 if not.

Using the assignment matrix *J*, we construct an *l* *df* MLC test statistic such that

(3) $$\begin{array}{c} G_{M}=\left(W_{}^{T}\hat{\beta }\right){\left(W_{}^{T}\Sigma W\right)}^{-1}\left({\hat{\beta }}^{T}W\right), \end{array}$$

where *W* = (Σ^−1^ *J*)(*J* ^*T*^Σ^−1^ *J*)^−1^ [8, 13].

If only one SNP is assigned to each cluster (singleton), *G*_*M*_ is the same as the generalized Wald test statistic

(4) $$\begin{array}{c} G_{W}={\hat{\beta }}_{}^{T}\Sigma^{-1}\hat{\beta }. \end{array}$$

Moreover, if all SNPs are assigned to one cluster, *G*_*M*_ is a linear combination (LC) test [1]. The asymptotic null distribution of the Wald test statistic is an *m* *df* chi-square distribution, assuming no linear dependencies among SNP genotypes, whereas the MLC test statistic follows an *l* *df* chi-square distribution. The asymptotic null distribution of the LC test statistic is 1 *df* chi-square.

### 2.2. Allele Recoding Algorithm

As shown in Yoo et al. [13], power of the LC and the MLC tests benefits from high positive correlation between causal and neutral SNPs or between causal SNPs. The sign of the correlation *r*_*ij*_ between two SNPs changes if we switch the risk and base alleles for one of the two SNPs. For example, if we replace *X*_*i*_ with a new genotype variable *X*_*i*_′ = 2 − *X*_*i*_, then the genotype of *X*_*i*_ = 0, 1, 2 becomes *X*_*i*_′ = 2, 1, 0, respectively, under an additive model. When this change is applied, the correlation between the genotype *X*_*i*_′ and *X*_*j*_ becomes −*r*_*ij*_ for *i* ≠ *j*. This coding change will also change the sign of beta estimates ${\hat{\beta }}_{i}$ if ${\hat{\beta }}_{i}\neq 0$. To make most pairwise correlations positive for SNPs in the joint analysis, we apply the Wang and Elston's SNP recoding algorithm [2], which is as follows.

**Step 1 .**

Count the number of negatively correlated SNPs for each SNP *i* and denote it as *n*_*i*_ for *i* = 1,2,…, *m*; that is, *n*_*i*_ = ∑_*j*=1,*j*≠*i*_^*m*^ *I*  (*r*_*ij*_ < 0), where *I* is an indicator function.

**Step 2 .**

Select the SNP with the max⁡{*n*_*i*_}; then switch the risk and base allele for the genotype of that SNP.

**Step 3 .**

Iterate Steps 1-2 with updated correlations from the updated genotypes until max⁡{*n*_*i*_} < *m*/2.

For the MLC test based on the LDSelect algorithm, we applied recoding for each cluster separately after clustering. With the CLQ algorithm, we incorporate recoding within the clustering algorithm.

### 2.3. SNP Clustering Using the LDSelect Algorithm

The LDSelect algorithm [19] is as follows.

**Step 1 .**

Set a threshold value *c* for correlation *r* between two SNPs. Suppose the *m* SNPs in a gene are indexed with *V*_1_ = {1,2,…, *m*}. Let *V*′ : = *V*_1_.

Starting with *B*_1_, iterate the selection of the *k*th cluster *B*_*k*_ in Steps 2 to 4.

**Step 2 .**

For each SNP *i* in *V*′, count the number of other SNPs having correlation with SNP *i* greater than a threshold value *c* such that *t*_*i*_ = ∑_*j*∈*V*′,*j*≠*i*_ *δ*_*ij*_, where

(5) $$\begin{array}{c} \delta_{ij}=\left\{\begin{array}{cc} 1 & \left|r_{ij}\right|>c\\ 0 & \text{otherwise}. \end{array}\right. \end{array}$$

We call the SNPs that meet this criteria the* neighbors* of SNP *i*. Proceed to Step 5 if *t*_*i*_ is at most equal to 0 for all *i* ∈ *V*′. If not, proceed to the next step.

**Step 3 .**

First, select one SNP (say *j*) with *t*_*j*_ = max⁡_*i*∈*V*′_⁡*t*_*i*_ and all the neighbors of SNP *j* in *V*′ and group them as *B*_*k*_ = {*i* ∈ *V*′ : |*r*_*ij*_ | >*c*}. When there is more than one SNP with the maximum number of neighbors, randomly select one SNP from among them.

**Step 4 .**

Remove SNPs in *B*_*k*_ from *V*_*k*_ and denote it as *V*_*k*+1_ = *V*_*k*_∖*B*_*k*_. Also, update *V*′ with *V*_*k*+1_. Iterate Steps 2~4 unless the condition to proceed to Step 5 is met or all SNPs are assigned into a cluster.

**Step 5 .**

If the maximum *t*_*i*_ for all *i* ∈ *V*′ is at most 0, the SNPs in *V*′ will be partitioned into singleton clusters (each with only one SNP).

*End*. In this way all the SNPs are assigned into clusters *B*_1_,…, *B*_*l*_ for some *l* ≤ *m*. Then *V*_1_ = ∪_*k*=1_^*l*^ *B*_*k*_, where *B*_*j*_∩*B*_*k*_ = *ϕ* for *j* ≠ *k* and *B*_*k*_ ≠ *ϕ* for *k* = 1,…, *l*.

### 2.4. SNP Clustering Using CLQ Algorithm

Let *G* = (*V*, *E*) be a graph with a vertex set *V* and an edge set *E* of *V*, the set of some pairs of vertices in *V*. If an edge between two vertices is included in *E*, we call these two adjacent. A* clique* is defined as a subset *C* of *V* such that all pairs of vertices in *C* are adjacent. A* maximal clique* in *G* is a clique whose vertices are not a subset of the vertices of a larger clique, and the* maximum cliques* in *G* are the largest among all cliques in a graph. A* subgraph* of *G* is a graph with a vertex set *V*′⊆*V* and an edge set *E*′⊆*E*. A subgraph *G*′ = (*V*′, *E*′) of *G* is said to be* induced* by a vertex set *V*′⊆*V* when an edge is in *E*′ if and only if the edge is in *E*. The subgraph induced by a clique is complete (all possible edges between vertices in clique are included in the edge set).

The CLQ algorithm is as follows.

**Step 1 .**

For a threshold value *c*, construct a graph *G*_1_ with a vertex set *V*_1_ = {1,2,…, *m*} corresponding to SNP 1,2,…, *m* in a gene and an edge set *E*_1_ in which the undirected edge between vertex *i* and *j* is included if |*r*_*ij*_ | >*c* for *i* ≠ *j*. Let *G*′ : = *G*_1_ with *V*′ : = *V*_1_ and *E*′ : = *E*_1_.

Starting with *B*_1_, iterate the selection of the *k*th cluster *B*_*k*_ in Steps 2 to 4.

**Step 2 .**

For each vertex in *G*′, find the maximal cliques that contain the vertex using the Bron-Kerbosch algorithm [20] implemented in igraph package [21] and select the largest clique of maximal cliques found for all vertices. Proceed to Step 5 if there is no maximum clique with at least two vertices. Otherwise, proceed to the next step.

**Step 3 .**

Apply the recoding algorithm to the maximum cliques chosen in Step 2. If all pairwise correlations between SNPs in the clique can be recoded to be positive, then take the SNPs corresponding the chosen clique as the cluster *B*_*k*_. If negatively correlated SNPs still exist after the recoding algorithm has been applied to this clique, discard the chosen clique and select the next largest one. If there are multiple cliques in *G*′ with the largest size and all SNPs can be recoded to be positively correlated, choose the one with the largest sum of absolute correlation. Repeat application of the recoding algorithm until *B*_*k*_ is determined. If there is no clique with at least two vertices that can be recoded to have all positive correlations, proceed to Step 5.

**Step 4 .**

Remove SNPs in *B*_*k*_ from *V*_*k*_ and denote it as *V*_*k*+1_ = *V*_*k*_∖*B*_*k*_. Also, update *V*′ with *V*_*k*+1_. Update *G*′ with the subgraph *G*_*k*+1_ induced by *V*_*k*+1_. The edge set is also updated by the edge set of *G*_*k*+1_ as *E*′ : = *E*_*k*+1_. Iterate Steps 2~4 unless the condition to proceed to Step 5 is met or all SNPs are assigned into a cluster.

**Step 5 .**

If there is no maximum clique with at least two vertices in *G*′, the SNPs in *V*′ will be partitioned into singleton clusters.

*End*. In this way all the SNPs are assigned into clusters *B*_1_,…, *B*_*l*_ for some *l* ≤ *m*. Then *V*_1_ = ∪_*k*=1_^*l*^ *B*_*k*_, where *B*_*j*_∩*B*_*k*_ = *ϕ* for *j* ≠ *k* and *B*_*k*_ ≠ *ϕ* for *k* = 1,…, *l*.

### 2.5. Comparison of Clustering Results

To compare the clusters produced for a gene by the two different clustering methods, we used the *S* criterion of Rand [22] and the *S*′ criterion that is adjusted for chance agreement [23]. Suppose in one clustering method, *m*, that SNPs are partitioned into *B*_1_,…, *B*_*l*_ and, using another method, they are partitioned into *C*_1_,…, *C*_*h*_. The similarity between two clustering results is defined as

(6) $$\begin{array}{c} S=\frac{\left(\sum_{i<j}^{m}a_{ij}\right)}{\left(\begin{array}{c} m\\ 2 \end{array}\right)}, \end{array}$$

where *a*_*ij*_ = 1 if there exist *k* and *k*′ such that SNP *i* and SNP *j* are both in *B*_*k*_ and *C*_*k*′_, or if there exist *k* and *k*′ such that SNP *i* is in both *B*_*k*_ and *C*_*k*′_ while SNP *j* is in neither *B*_*k*_ nor *C*_*k*′_, and *a*_*ij*_ = 0 otherwise. A higher value of *S* corresponds to more similar performance of two clustering methods for the given data. When a pair of clustering results are exactly identical, then *S* = 1, whereas if *S* = 0 there is no similarity. The adjusted agreement measure *S*′ is defined as

(7) S′=∑i,jmij2−∑imi·2∑jm·j2/m21/2∑imi·2+∑jm·j2−∑imi·2∑jm·j2/m2,

where *m*_*ij*_ denotes the number of common SNPs that belong to clusters *B*_*i*_ and *C*_*j*_, and *m*_*i*·_ and *m*_·*j*_ are the number of SNPs in clusters *B*_*i*_ and *C*_*j*_, respectively [23].

### 2.6. Numerical Power Analysis Based on HapMap Data

Based on 1000 gene structures obtained from HapMap phase III Asian data, we computed MLC test power using LDSelect clustering (MLC-LD) and CLQ clustering (MLC-CL) for several alternative trait models with one or two causal SNPs. The HapMap gene panels were obtained by random selection from the 8883 genes that had 4~30 SNPs, excluding SNPs with MAF < 0.01 or any pairwise LD value |*r*| > 0.99. Two sets of 1000 genes were randomly selected, allowing overlap: one for the analysis of Models A, B, and C and another for validation analysis. For each set of 1000 genes, two panels of SNPs with MAF ≥ 0.05 and MAF ≥ 0.01, respectively, were used in comparisons of clustering results, and one panel with MAF ≥ 0.01 was used for power analysis. We evaluated a range of clustering threshold values for *c* equal to 0.3 through 0.9 for LDSelect and CLQ.

For trait models, we considered models with one to four causal SNPs within a gene and a linear model for the quantitative phenotype *Y*:

(8) $$\begin{array}{c} Y=\overset{t}{\underset{i=1}{\sum }}b_{i}G_{i}+\varepsilon , \end{array}$$

where *t* is the number of causal SNPs, *b*_*i*_ is the effect of *i*th causal SNP, *G*_*i*_ is the number of causal alleles for the *i*th causal SNP, and *ε* is the error term assumed to follow a normal distribution with mean 0 and variance *σ* ^2^ (Table 1).

Initially we considered three types of trait models: one with one causal SNP in a gene with effect *b*_1_ = 1 (Model A), another with two causal SNPs in a gene with effects *b*_1_ = 1, *b*_2_ = 1 (Model B), and a third with two causal SNPs in a gene with effects *b*_1_ = 1, *b*_2_ = −1 (Model C). Since power in a linear model depends on the ratio of signal to noise, that is, (*b*_*i*_/*σ*), we selected the variance *σ* ^2^ to correspond to reasonable power for a given gene structure and choice of causal SNPs for Models A, B, and C. To clearly see relative performance of the MLC tests compared to the generalized Wald test, we adjusted *σ* ^2^ such that the Wald test power is 60% for each trait model. For Model A, in each gene we chose each of the SNPs in turn to be the causal SNP, resulting in over 11,000 trait model settings in total over 1000 genes. For Models B and C, in each gene we chose each of all possible SNP pairs in turn to be the causal SNP pair, resulting in nearly 80,000 trait model settings in total for main and validation sets.

In a fourth trait model (Model D), we also obtained power over mixed types of causal models with variable effect sizes. The number of causal SNPs was chosen randomly between 1 and 4, with the deleterious and protective causal SNPs randomly assigned. For each causal SNP, |*b*_*i*_| was randomly selected from the uniform distribution *U*(0.01 × SD, 0.05 × SD) where SD is the expected standard deviation of *Y* following the effect size estimates for SNPs associated with lipid levels presented in Willer et al. [24]. Then the error variance *σ* ^2^ was fixed as 1.

For power analysis using Models A to D, the genotype data were randomly generated from the haplotype panel of HapMap data for *n* = 5,000 subjects. Power was calculated numerically for each gene assuming asymptotic chi-square distributions under the null and alternative trait models. With a given significance level *α* and number of clusters *l*, the critical value *c*_*l*,*α*_ is obtained from the *l* *df* chi-square distribution such that *P*{*χ*_*l*_^2^ > *c*_*l*,*α*_} = *α*. Power is computed as *P*{*χ*_*l*,*λ*_^2^ > *c*_*l*,*α*_} with *l* *df* and noncentrality *λ* parameter equal to the expected MLC statistic value under the trait model (see Appendix in [13]).

## 3. Results

### 3.1. Comparison of SNP Clustering by LDSelect and CLQ

**Figure 1:**
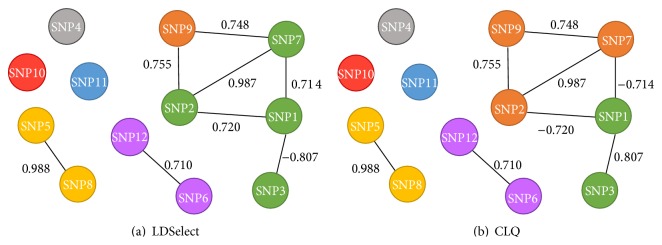
Clustering of gene ARHGAP29 by LDSelect and CLQ for a threshold value 0.7.

In Figure 1, we illustrate LDSelect and CLQ clustering for 12 SNPs in ARHGAP29 at a threshold value 0.7. By LDSelect, the largest cluster includes SNPs 1, 2, 3, and 7 since SNP 1 tags SNPs 2, 3, and 7. However with CLQ, these four SNPs do not form a clique because the pairwise correlations between SNPs 2 and 3 and between SNPs 3 and 7 are below the threshold value. By CLQ, SNPs 2, 7, and 9 are clustered as a clique and SNPs 1 and 3 are clustered as another clique. Here, SNP 1 is recoded so that the correlation within the clique is positive.

We compared LDSelect and CLQ clustering for each of the 1000 HapMap genes across a range of threshold values. For a given threshold, the clustering methods often produce identical gene clusters, particularly at higher threshold values (Table 2). For example, at the threshold value 0.7, 54% of the clustering results are the same. With increased threshold values, the averages of the agreement measures *S* and *S*′ also increase. At threshold values greater than 0.5, the average agreement measure *S*′ is greater than 78% overall. Comparison of average *S* and *S*′ under stratification by five gene-size groups (≤10, 11~15, 16~20, 21~25, >25 SNPs per gene) showed that the agreement slightly weakens with increased number of SNPs (results not shown).

**Figure 2:**
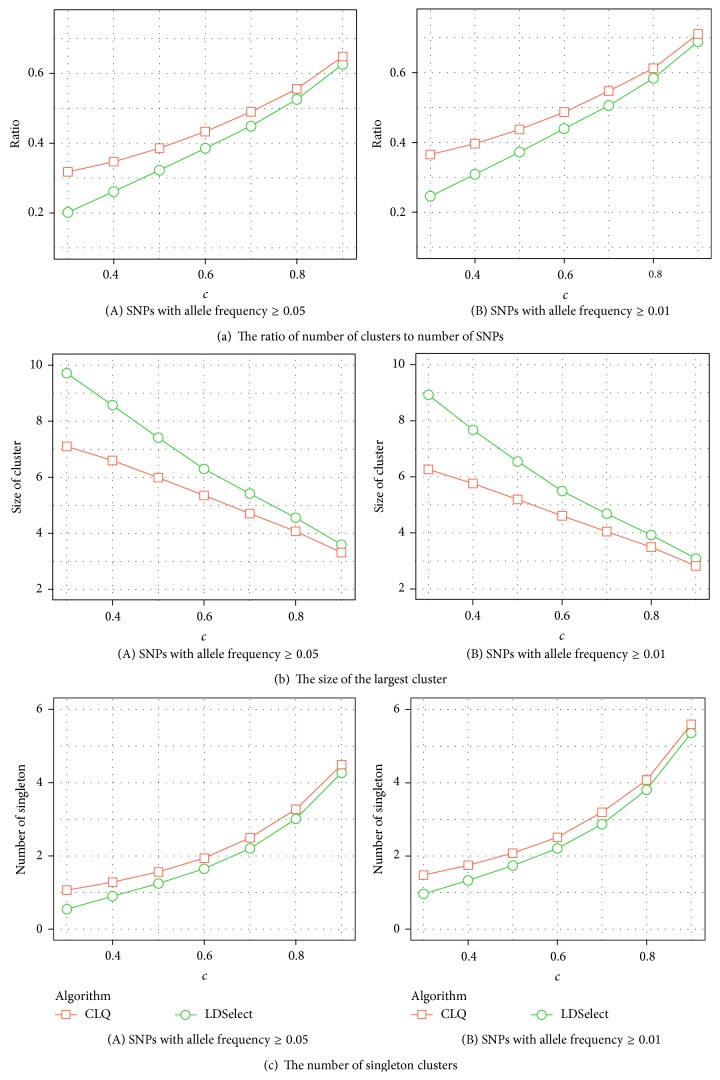
Averages of (a) the ratio of number of clusters to number of SNPs, (b) the size of the largest cluster, and (c) the number of singleton clusters in each of 1000 genes for LDSelect and CLQ clustering given a threshold value *c*.

On average, the number of clusters obtained by LDSelect is usually smaller than that by CLQ for a given threshold value (Table 3). Cluster sizes are smaller and less variable in CLQ than in LDSelect, averaged over all gene sizes (Table 3).  Figure 2(a) shows the average over 1000 genes of the ratio of the number of clusters to the number of SNPs per gene used for clustering by LDSelect and CLQ given a threshold value *c*. Restricting the SNPs to have higher minor allele frequency (MAF ≥ 0.05 versus MAF ≥ 0.01) reduces the ratio similarly for both clustering methods. At the same threshold value, CLQ produces a larger number of clusters compared to LDSelect, mainly because CLQ has a stricter within-cluster LD requirement, but this difference decreases as the threshold value increases. It follows that the average size of the largest cluster in each gene is greater for LDSelect than for CLQ (Figure 2(b)), with greater differences at lower threshold values. Conversely, CLQ usually produces more singleton clusters than LDSelect (Figure 2(c)). We conclude that at the same threshold value, CLQ tends to produce more clusters of smaller size than LDSelect.

**Figure 3:**
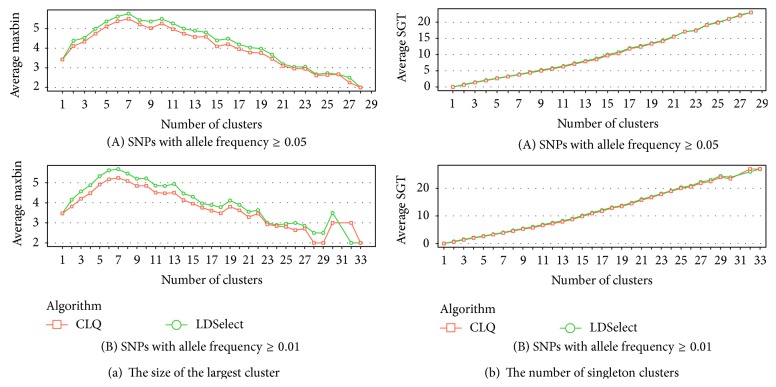
Averages of (a) the size of the largest cluster and (b) the number of singleton clusters produced in each gene by LDSelect and CLQ for a fixed number of clusters per gene. For each gene, the number of clusters produced by each clustering method was found at threshold values within a grid from 0.1 to 0.9 by 0.01. Excluding results at the extremes (i.e., including cluster numbers that fell between 20% and 90% of the number of SNPs), the LDSelect and CLQ cluster numbers were matched for each gene and the maximum cluster size for each was averaged across genes at a fixed value of the number of clusters.

To compare maximum cluster size when the number of clusters per gene is the same, rather than at a fixed threshold value, we applied the clustering methods across a range of threshold values and for each gene matched the LDselect and CLQ clustering results according to the number of clusters (Figure 3(a)). At nearly all cluster numbers, the average size of the largest cluster is again smaller for CLQ. Out of all clustering results with different numbers of clusters, 69% had the same maximum cluster size by LDSelect and CLQ, 23% had a larger maximum cluster by LDSelect, and only 8% had a smaller maximum cluster size by LDSelect, based on SNPs with MAF ≥ 0.05. For the SNPs with MAF ≥ 0.01, these percentages were 65%, 28%, and 7%. For a fixed number of clusters, the number of singleton clusters was slightly smaller for CLQ than LDSelect (Figure 3(b)). Out of all clustering results, 70% had the same number of singleton clusters by LDSelect and CLQ, 21% had a larger number by LDSelect, and only 9% had a smaller number of singleton clusters by LDSelect than CLQ, based on SNPs with MAF ≥ 0.05. For the SNPs with MAF ≥ 0.01, these percentages were 67%, 25%, and 8%. We conclude that when the two clustering methods produce the same number of clusters for a gene, the CLQ clusters will tend to be less variable in size than the LDSelect clusters. We draw similar conclusions from the analysis of validation set (results not shown).

### 3.2. Comparison of MLC-LD and MLC-CL Test Power

For the power calculations, the variance of the error term was set such that Wald test power is 60% for Models A, B, and C. For Model D, the variance was fixed as 1 and variation in the regression coefficient determined power. In Table 4, the average MLC test power values for trait model types A, B, C, and D using two clustering methods (MLC-LD and MLC-CL) vary across values of the clustering threshold *c*. When averaged over all sets of genes and causal SNP choices, MLC-LD power and MLC-CL power were both higher than Wald test power (which was 60% for Models A–C and roughly 35–38% for Model D). Average power was usually maximized at *c* = 0.6 or 0.7 for LDSelect and at *c* = 0.4 or 0.5 for CLQ. At threshold values less than 0.7, MLC-CL power was higher than MLC-LD for all models. At threshold values higher than 0.6, however, MLC-CL power was less than MLC-LD for Models A, B, and D. For Model C, MLC-CL power was higher than MLC-LD for all threshold values. For each model, the highest average power was achieved by MLC-CL (bolded entries in Table 4). Comparison of average MLC power values under stratification by five gene-size groups (≤10, 11~15, 16~20, 21~25, >25 SNPs per gene) generally yielded similar results (results not shown).

**Figure 4:**
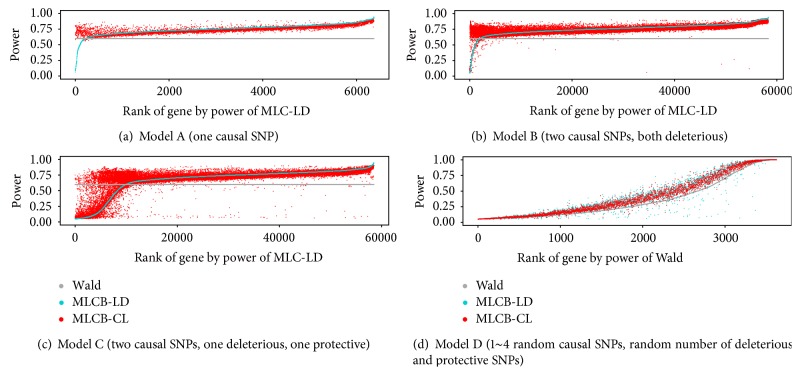
Power of MLC test based on LDSelect clustering (MLC-LD: blue) and CLQ clustering (MLC-CL: red) with threshold *c* = 0.7 for the cases where LDSelect and CLQ clusters are different. Each point represents one case of all possible causal SNP assignments within a gene. The variance of the error term was adjusted for each of 1000 genes such that the Wald test power is exactly 0.6 (Grey) for Models A, B, and C and fixed as 1 for Model D.

We also compared the proportion of gene-causal-SNP cases in which MLC-LD power or MLC-CL power was less than Wald test power (Table 4). The proportion with lower MLC-CL power was smaller, suggesting improved robustness. At lower threshold values particularly, the proportion with power less than the Wald test for LDSelect was much higher, up to 40~54% for some models, but was less than 25% for CLQ. Plots of gene-specific power obtained for the cases in which the LDSelect and CLQ clusters differ show that the MLC test using CLQ is less likely than MLC test using LDselect to have substantially reduced power relative to the Wald test (Figure 4). Similar conclusions about relative power were obtained from the analysis of validation set (results not shown).

## 4. Discussion

In previous studies, we reported that power of the MLC test depends on the correlation structure among SNPs and we postulated that clusters of strongly correlated SNPs with positive correlations benefit the test [13]. Therefore, the CLQ algorithm designed to construct such clusters, in which all pairwise correlations in the cluster are positive and strong, should work well for MLC tests. LDSelect and CLQ produced exactly identical clusters for about 38~54% of the genes at threshold values *c* = 0.5~0.7. This implies that many of the clusters found by LDSelect using a SNP that tags other SNPs are actually cliques; that is, the pairwise correlations between SNPs in the cluster other than the SNP with most neighbors are also above the threshold, even though the LDSelect algorithm does not consider that information.

The LDSelect algorithm was originally developed for tag SNP selection so that indirect associations could be efficiently captured by genotyping and analyzing only tag SNPs. We observed that the LDSelect algorithm also works reasonably well for MLC tests where power depends on formation of the clusters with large positive correlations. Because the algorithms produce identical clusters for a substantial portion of the cases, the average MLC test power values were not dramatically different over the genes we tested. However, robustness as measured by the proportion of gene-causal-SNP cases with lower power than the Wald test was better with CLQ than with LDSelect. The plot of entire power values for all trait-causal SNP models over 1000 genes also indicated that the MLC test using CLQ is less likely than LDSelect to have substantially reduced power relative to the Wald test. Because the CLQ algorithm produces slightly more clusters than LDSelect for a given threshold, the degrees of freedom tends to be higher for the MLC test using CLQ than LDSelect, and in that matter CLQ has a disadvantage compared to LDSelect. However, the smaller sized clusters constructed by CLQ may be advantageous because SNPs with opposing effects are less likely to occur in the same cluster.

For power comparisons using different threshold values, we found that CLQ with threshold values *c* = 0.4~0.5 usually produces the best average power among all clustering algorithm-threshold value combinations. In previous studies [8, 13], we suggested the threshold value 0.3~0.5 for *r* ^2^ (0.5~0.7 for |*r*| ≤ *c*) to achieve optimum power using LDSelect algorithm, which has been validated by the results of this study. We suggest the threshold value of *c* = 0.4~0.5 to be used for CLQ algorithm based on the results of this study. However, a dynamically determined threshold value after evaluating the LD structure might be more appropriate for MLC tests, and being able to choose nonarbitrary threshold values is more attractive to researchers applying the method.

We applied the CLQ algorithm to a prespecified gene unit for gene-based analysis, but it could be applied similarly to intergenic regions, exomes, and promoter regions, that is, any regional units exhibiting some linkage disequilibrium between SNPs. If these regions include too many SNPs (e.g., more than 100), it is unreasonable to apply MLC tests based on joint regression models unless the sample size is extremely large. In that case, it may be desirable to break up the region into several LD blocks. Another approach would be to apply variable selection techniques such as penalized regressions [25, 26] and construct a MLC-type test with the resulting models.

## 5. Conclusions

In summary, we observed that CLQ and LDSelect produce identical clusters about half the time, and in the remaining cases, CLQ usually produces more clusters of smaller size. On average, MLC test power using CLQ is similar to that using LDSelect. The MLC test using CLQ shows better robustness to the detrimental effects of opposing SNP associations within the same cluster. Therefore, the CLQ algorithm is a promising approach for preanalysis clustering of SNPs for multimarker methods such as the MLC test.

## Figures and Tables

**Table 1:** Quantitative trait models used for power comparisons of MLC-LD and MLC-CL.

Model name	Description	Trait model parameters^*^
Model A	One causal SNP within a gene	*b*_1_ = 1

Model B	Two causal SNPs, both deleterious	*b*_1_ = 1, *b*_2_ = 1

Model C	Two causal SNPs, one deleterious and one protective	*b*_1_ = 1, *b*_2_ = −1

Model D	1~4 causal SNPs, random assignment of the direction of effects	|*b*_*i*_| is randomly selected from the *U*(0.01 × SD, 0.05 × SD) where SD is the expected standard deviation of *Y*

^*^The trait model is *Y* = ∑_*i*=1_^*C*^ *b*_*i*_ *G*_*i*_ + *ε* where *ε* ~ *N*(0, *σ* ^2^), *C* is the number of causal SNPs, *b*_*i*_ is the effect of *i*th causal SNP, and *G*_*i*_ is the number of causal alleles for the *i*th causal SNP. The variance σ^2^ is adjusted to make the power of Wald test 60% for each set of causal SNPs for Models A, B, and C and set to 1 for Model D.

**Table 2:** Mean and standard deviation over 1000 genes of agreement measure *S* and *S*′ for two clustering methods (LDSelect and CLQ) and number of genes with identical clustering.

Allele frequency cut	*c*	*S*		*S*′		Cases of perfect agreement
		Mean	SD	Mean	SD	
.05	.3	.676	0.203	.325	0.342	180
	.4	.769	0.191	.510	0.336	283
	.5	.847	0.168	.665	0.303	388
	.6	.909	0.123	.781	0.242	483
	.7	.936	0.101	.832	0.210	541
	.8	.959	0.086	.884	0.178	648
	.9	.974	0.069	.918	0.156	736

.01	.3	.689	0.196	.395	0.376	155
	.4	.789	0.177	.559	0.379	254
	.5	.863	0.151	.687	0.338	361
	.6	.923	0.105	.794	0.267	468
	.7	.948	0.084	.843	0.230	536
	.8	.968	0.068	.892	0.201	644
	.9	.981	0.053	.922	0.172	744

**Table 3:** The average over 1000 genes of the number of clusters per gene, the mean size of the clusters within a gene, and the standard deviation of the cluster sizes within a gene for two clustering methods (LDSelect and CLQ).

Allele frequency cut	*c*	# of clusters^*^		Mean size of clusters^*^		SD size of clusters^*^	
		LDSelect	CLQ	LDSelect	CLQ	LDSelect	CLQ
.05	.3	1.84	2.94	6.39	3.70	*2.55 *	*2.60 *
	.4	2.43	3.27	4.82	3.40	2.82	2.33
	.5	3.02	3.67	3.85	3.06	2.49	2.02
	.6	3.65	4.19	3.13	2.67	2.13	1.75
	.7	4.36	4.79	2.61	2.32	1.75	1.46
	.8	5.18	5.52	2.18	2.01	1.38	1.19
	.9	6.29	6.57	1.76	1.65	1.00	0.89

.01	.3	2.40	3.53	5.25	3.28	3.33	2.53
	.4	3.02	3.91	4.08	3.00	3.02	2.25
	.5	3.66	4.36	3.32	2.71	2.52	1.96
	.6	4.35	4.90	2.75	2.40	2.07	1.67
	.7	5.10	5.57	2.34	2.10	1.66	1.40
	.8	5.99	6.32	1.98	1.85	1.31	1.15
	.9	7.17	7.42	1.64	1.56	0.94	0.85

^*^The differences of the obtained characteristics within genes are compared by paired *t*-test and all results were significant with *P* values <1*e* ^−10^ except the italic pairs (*P* = 0.61).

**Table 4:** Average MLC test power over all gene-causal-SNP combinations for LDSelect (MLC-LD) and CLQ (MLC-CL) clustering methods and the proportion of genes where MLC-LD power and MLC-CL power are less than Wald test power.

Model	*c*	All possible causal SNPs and all genes					All possible causal SNPs for the genes where LDSelect and CLQ clusters are different				
		*N*	Average^†,∗^		% Power < Wald^*^		*N*	Average^†,∗^		% Power < Wald^*^	
			LDS	CLQ	LDS	CLQ		LDS	CLQ	LDS	CLQ
A	0.3	11,117	0.627	0.757	36.6	6.2	9,765	0.614	0.759	40.0	5.8
	0.4	11,117	0.670	**0.758 **	26.4	3.9	8,867	0.656	**0.762 **	30.5	3.3
	0.5	11,117	0.716	0.754	14.6	2.2	8,069	0.714	0.759	17.2	1.8
	0.6	11,117	**0.735 **	0.745	6.7	1.0	7,381	0.742	0.753	8.1	0.7
	0.7	11,117	0.733	0.730	2.7	0.6	6,234	**0.751 **	0.744	3.4	0.2
	0.8	11,117	0.719	0.712	1.1	0.6	5,138	0.746	0.731	0.8	0.0
	0.9	11,117	0.691	0.685	1.4	1.3	3,512	0.726	0.707	0.3	0.0

B	0.3	79,650	0.645	0.771	33.7	5.6	74,715	0.640	0.774	35.0	5.2
	0.4	79,650	0.682	**0.773 **	25.5	3.6	70,384	0.674	**0.775 **	27.3	3.0
	0.5	79,650	0.727	0.769	14.5	2.1	66,788	0.723	0.770	15.8	1.7
	0.6	79,650	**0.750 **	0.760	6.4	1.2	63,848	0.752	0.764	7.0	0.9
	0.7	79,650	0.748	0.745	3.0	0.6	57,300	**0.756 **	0.752	3.5	0.5
	0.8	79,650	0.733	0.724	0.9	0.4	48,577	0.752	0.737	0.7	0.2
	0.9	79,650	0.701	0.692	0.9	0.5	33,403	0.724	0.706	0.8	0.1

C	0.3	79,650	0.499	0.649	54.3	23.7	74,710	0.505	0.663	54.2	21.9
	0.4	79,650	0.551	0.657	44.1	21.1	70,409	0.557	0.675	44.0	18.6
	0.5	79,650	0.603	0.662	32.8	18.4	66,772	0.615	**0.683 **	32.0	15.5
	0.6	79,650	0.637	**0.664 **	23.7	16.4	63,910	0.651	0.682	22.8	14.1
	0.7	79,650	0.652	0.662	18.1	14.1	57,409	0.669	0.682	17.4	12.2
	0.8	79,650	**0.654 **	0.657	14.1	11.7	48,669	**0.675 **	0.678	13.8	10.3
	0.9	79,650	0.645	0.646	10.3	8.8	33,625	0.661	0.662	11.6	8.3

D^**^	0.3	8,883	0.388	0.444	36.5	12.1	7,054	0.372	**0.441 **	39.7	9.9
	0.4	8,883	0.408	0.447	28.3	9.7	6,140	0.389	0.440	32.5	7.3
	0.5	8,883	0.426	**0.447 **	18.8	7.4	5,119	0.404	0.433	22.1	5.0
	0.6	8,883	**0.439 **	0.445	10.8	5.5	4,420	**0.425 **	0.435	12.5	3.5
	0.7	8,883	*0.439 *	*0.440 *	6.5	4.2	3,625	*0.416 *	*0.414 *	7.4	3.0
	0.8	8,883	0.435	0.433	4.4	3.3	2,827	0.419	0.412	4.1	1.7
	0.9	8,883	0.425	0.423	3.6	3.3	2,103	0.406	0.396	2.7	1.7

^*^The differences of power between two clustering algorithm and the proportions of cases with MLC test power less than the power of Wald test within genes are compared by paired *t*-test and McNemar test, respectively, and all results are significant with *P* values <0.05 except the italic pairs.

^**^The power of Wald test for Models A, B, and C were fixed as 0.6, whereas the average power of Wald test for Model D was 0.388 in average over all genes (left) and 0.377, 0.373, 0.365, 0.368, 0.354, 0.356, and 0.351 for *c* = 0.3 ~ 0.9, respectively, for genes with clustering results are different (right).

^†^Bolded numbers are the maximum average power of MLC over different threshold values within each clustering method, trait model, and the set of genes (all or the ones with different clustering results by LDSelect and CLQ).
